# Peptidyl tRNA Hydrolase Is Required for Robust Prolyl-tRNA Turnover in Mycobacterium tuberculosis

**DOI:** 10.1128/mbio.03469-22

**Published:** 2023-01-25

**Authors:** Francesca G. Tomasi, Jessica T. P. Schweber, Satoshi Kimura, Junhao Zhu, Laura A. T. Cleghorn, Susan H. Davis, Simon R. Green, Matthew K. Waldor, Eric J. Rubin

**Affiliations:** a Department of Immunology and Infectious Diseases, Harvard T. H. Chan School of Public Health, Boston, Massachusetts, USA; b Division of Infectious Diseases, Brigham and Women’s Hospital, Boston, Massachusetts, USA; c Department of Microbiology, Harvard Medical School, Boston, Massachusetts, USA; d Howard Hughes Medical Institute, Boston, Massachusetts, USA; e Drug Discovery Unit, Wellcome Centre for Anti-Infectives Research, Division of Biological Chemistry and Drug Discovery, College of Life Sciences, University of Dundee, Dundee, United Kingdom; The Hebrew University of Jerusalem

**Keywords:** *Mycobacterium tuberculosis*, antibiotic resistance, bacterial genetics, ribosomes, tRNA, tRNA sequencing, translation

## Abstract

Enzymes involved in rescuing stalled ribosomes and recycling translation machinery are ubiquitous in bacteria and required for growth. Peptidyl tRNA drop-off is a type of abortive translation that results in the release of a truncated peptide that is still bound to tRNA (peptidyl tRNA) into the cytoplasm. Peptidyl tRNA hydrolase (Pth) recycles the released tRNA by cleaving off the unfinished peptide and is essential in most bacteria. We developed a sequencing-based strategy called copper sulfate-based tRNA sequencing (Cu-tRNAseq) to study the physiological role of Pth in Mycobacterium tuberculosis (Mtb). While most peptidyl tRNA species accumulated in a strain with impaired Pth expression, peptidyl prolyl-tRNA was particularly enriched, suggesting that Pth is required for robust peptidyl prolyl-tRNA turnover. Reducing Pth levels increased Mtb’s susceptibility to tRNA synthetase inhibitors that are in development to treat tuberculosis (TB) and rendered this pathogen highly susceptible to macrolides, drugs that are ordinarily ineffective against Mtb. Collectively, our findings reveal the potency of Cu-tRNAseq for profiling peptidyl tRNAs and suggest that targeting Pth would open new therapeutic approaches for TB.

## INTRODUCTION

Enzymes involved in ribosome rescue are ubiquitous in bacteria (1). mRNA truncation, frameshifting, and readthrough errors can result in unproductive ribosomes that stall on transcripts because the stop codon at the end of the mRNA is missing or no longer in frame (2). Certain amino acid combinations or codon patterns can also interfere with ribosome efficiency and lead to stalling (3). Even in the absence of cellular stressors, hiccups in protein synthesis occur with a sufficient frequency that the accumulation of stalled ribosomes would impair cell survival (4).

To rescue stalled ribosomes, bacteria use a diverse set of pathways (5), which all result in stalled ribosomes being returned to the free pool of actively translating ribosomes. One mechanism of freeing the ribosome is peptidyl tRNA drop-off, or simply “drop-off,” where peptidyl tRNA dissociates from the ribosome either spontaneously or with the help of ribosome recycling factor (RRF), elongation factor G (EF-G), and release factor 3 (RF3) (6–8). A key distinction in drop-off is the release of peptidyl tRNA into the cytoplasm; in contrast, rescue systems like *trans*-translation and alternative rescue factors ArfA/B hydrolyze peptidyl tRNA that is still bound to the ribosome using different enzymes (1).

The central enzyme in peptidyl tRNA drop-off is peptidyl tRNA hydrolase (Pth), an esterase that recycles tRNA by cleaving the released peptidyl tRNA (7). While the structure of Pth has been solved in multiple organisms and its catalytic mechanism of action is well understood, little is known about the functional roles of peptidyl tRNA drop-off in bacteria *in vivo* (9, 10). tRNA turnover is complex and tightly regulated in cells (11), and Pth’s role in tRNA dynamics is not well understood. Pth does not have differential activity on different tRNA species *in vitro* (12–14), but it is unclear whether this enzyme preferentially cleaves specific tRNA isoacceptors *in vivo*.

Here, we examine the physiological role of Pth in Mycobacterium tuberculosis (Mtb), a pathogen that was responsible for over 10.5 million active cases of tuberculosis (TB) and at least 1.5 million deaths in 2020 (15). Mtb has been shown to be highly susceptible to the inhibition of *trans*-translation during normal growth, suggesting that this pathogen has a critical need for ribosome rescue machinery (16). Work on *trans*-translation in Mtb has opened doors for drug discovery efforts aimed at targeting ribosome rescue in Mtb (17). However, peptidyl tRNA drop-off and the effects of Pth depletion on Mtb have not yet been investigated. Here, we have developed copper sulfate-based tRNA sequencing (Cu-tRNAseq) to profile Mtb peptidyl tRNAs and study Pth in Mtb. We show that while peptidyl tRNA accumulates across most charged tRNA species in a Pth hypomorph, peptidyl prolyl-tRNA overtakes proline tRNA pools in a Pth knockdown construct, suggesting that Pth is required for robust prolyl-tRNA turnover. Reducing Pth levels increased Mtb’s susceptibility to tRNA synthetase inhibitors and macrolides, antibiotics that are not currently used to treat TB. Our work underscores the importance of Pth in tRNA turnover and opens new avenues for anti-TB strategies that exploit the synergy between translation errors and tRNA turnover.

## RESULTS

### *pth* is required for normal growth of Mtb.

**FIG 1:**
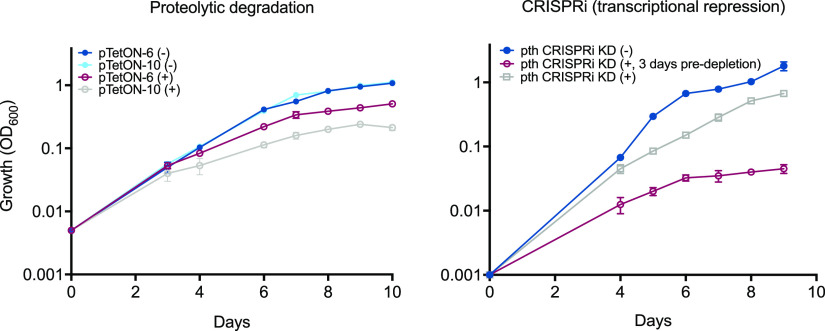
Complementary techniques show that Pth is required for normal growth in Mtb. Growth curves are shown for different Pth knockdown (KD) constructs: proteolytic degradation (left) and transcriptional repression (right). In both panels, “(−)” indicates uninduced and “(+)” indicates induced for Pth knockdown. Growth was measured spectrophotometrically for each strain with OD_600_ measurements over the course of 10 days in the presence or absence of an inducer. Prior to taking measurements, strains were diluted to the starting OD_600_ indicated at day 0. Numbers next to the plasmid names indicate the promoter strengths for SspB expression (6 is lower and 10 is higher, corresponding to the expected relative levels of protein depletion). “pre-depletion” refers to growing cells in the presence of aTC for 3 to 4 days prior to diluting strains to the indicated starting OD_600_s. Predepletion enables a higher level of *pth* knockdown to be achieved before conducting experiments. The results for both graphs are from two biological replicates.

Transposon sequencing in Mtb suggests that, like in Escherichia coli, the gene encoding Pth (Rv1014c) is required for survival (18, 19). We constructed two types of Mtb Pth mutants using complementary genetic techniques: proteolytic degradation and CRISPR interference (CRISPRi). While the first approach depletes intracellular Pth levels by tagging proteins for degradation using a titratable system (20), the other achieves knockdown through transcriptional repression of various strengths based on the protospacer-adjacent motif (PAM) used to target catalytically dead Cas9 (dCas9) to *pth* (21). Both types of strains had growth defects that were proportional to the level of depletion (Fig. 1), confirming that Pth is required for the normal growth of Mtb. Due to the technical ease of validating *pth* knockdown levels and our use of the tightly regulated transcriptional repression strain in previous work (22), we used the CRISPRi depletion strain for subsequent experiments in this work.

We previously characterized a second function for Pth in Mtb. In addition to its peptidyl tRNA hydrolase activity, Pth is a detoxifying enzyme for a tRNA-acetylating toxin, TacT, that is part of a newly identified toxin-antitoxin system (22). We found that this function does not contribute to *pth* essentiality in Mtb since TacT was not active under normal laboratory growth conditions, and a *tacAT* knockout strain was equally susceptible to *pth* depletion by CRISPRi (22). Thus, we focused our efforts on studying the effects of Pth depletion in Mtb on peptidyl tRNA pools and tRNA turnover following translation errors.

### Cu-tRNAseq measures peptidyl tRNA levels.

**FIG 2:**
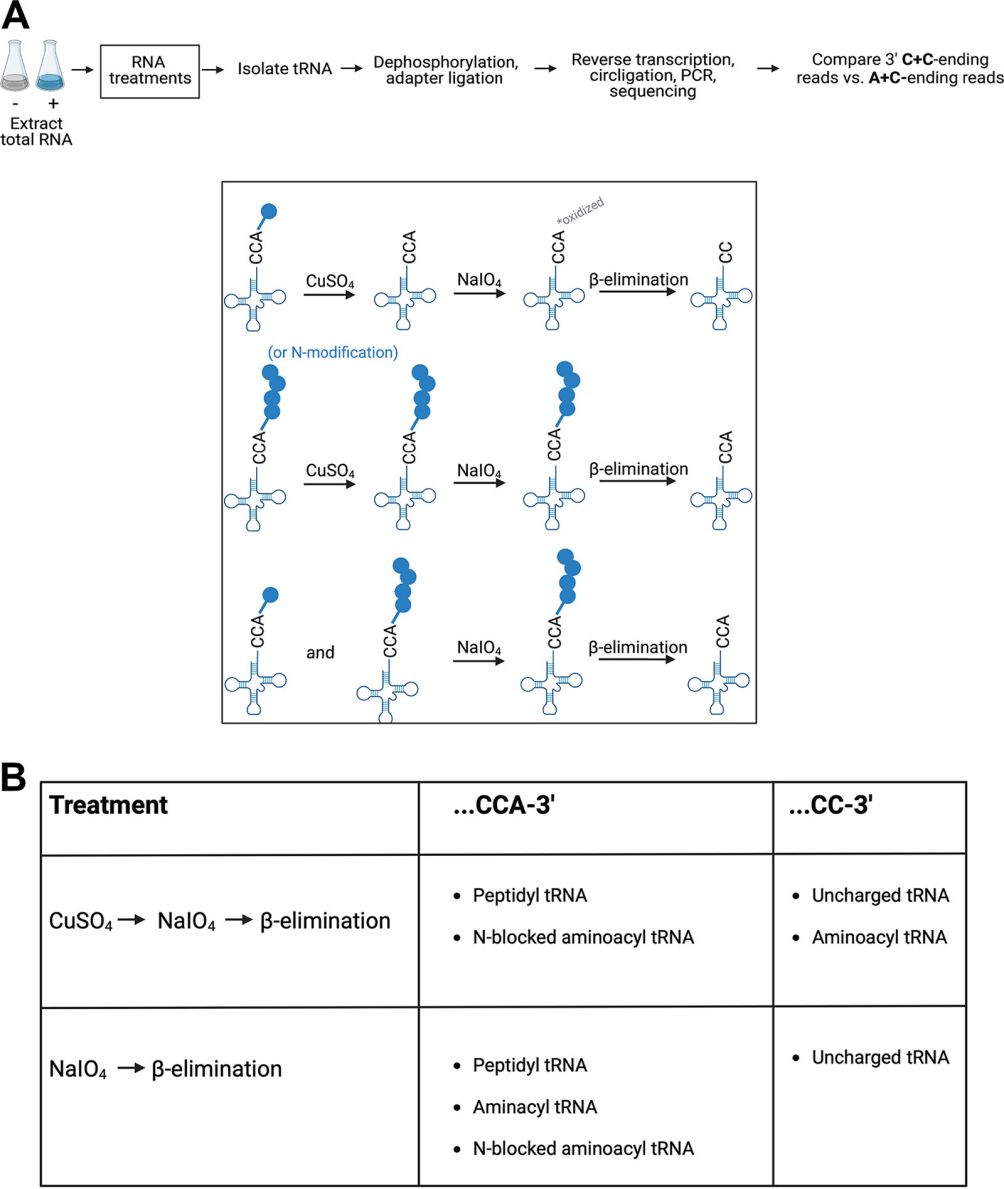
Application of tRNA sequencing to measure pools of peptidyl and N-acetylated tRNAs. (A) Overview of the copper sulfate-based tRNA sequencing (Cu-tRNAseq) protocol. Strains are grown and harvested at a normalized cell density. Total RNA is subjected to chemical treatments (boxed) to distinguish between aminoacyl or uncharged tRNA and peptidyl or N-blocked (e.g., N-acetylated) aminoacyl tRNA (top and middle, respectively). A control treatment is performed for each replicate without CuSO_4_ to measure total charged tRNA levels (bottom). tRNA fractions are extracted from 1 to 2 μg of total RNA for sequencing library preparation as described in Materials and Methods (26). (B) The ratio of 3′ A+C-ending reads to the total 3′ C+C- and 3′ A+C-ending reads for each tRNA is computed as described previously (27) to estimate the fraction of peptidyl (or N-acetylated) tRNA of each tRNA species. A higher fraction of A+C-ending reads following Cu-tRNAseq suggests a higher proportion of peptidyl or N-acetylated aminoacyl tRNA. (Figure created with BioRender.com.)

Studies of E. coli have shown that Pth depletion leads to the accumulation of peptidyl tRNA in cells (13). The identification of peptidyl tRNAs has typically been accomplished using Northern blotting (23), making it technically difficult to compare pools across all tRNA species and their respective isoacceptors. More recent work on other substrates for Pth such as N-acetylated aminoacyl tRNAs has surveyed tRNA species using mass spectrometry, which substantially increases sensitivity but still poses scalability challenges (22, 24, 25). We developed a tRNA sequencing-based strategy, dubbed Cu-tRNAseq, to study the effects of *pth* depletion on peptidyl tRNA profiles in a rapid, quantitative, and high-throughput manner (Fig. 2).

tRNA sequencing has been used to survey tRNA levels and modifications in both bacteria and eukaryotes, and recently, an application was developed to measure aminoacyl tRNA fractions (26). This method incorporates chemical treatments prior to sequencing that distinguish between charged and uncharged tRNAs. All tRNAs share a 3′ CCA tail that serves as the aminoacylation site (27). The first chemical treatment, oxidation by sodium periodate (NaIO_4_), has no effect on the 3′ end of a charged tRNA because the bound amino acid protects molecules from oxidation. However, an uncharged tRNA is oxidized at the 3′ A. A subsequent β-elimination reaction at high pH then removes an unprotected 3′ A residue at the end of uncharged tRNA or simply deacylates charged tRNA, leaving the 3′ A intact on the molecule. Sequencing of tRNA libraries and comparison of CC-ending reads to CCA-ending reads for each tRNA isoacceptor provide a global view of tRNA molecules that are protected by single amino acids or peptides at their 3′ ends (26).

We adapted this technique to monitor peptidyl tRNAs by adding a chemical treatment with copper sulfate (CuSO_4_) prior to periodate oxidation (Fig. 2). Incubation with CuSO_4_ deacylates tRNAs charged with a single amino acid, but this treatment leaves N-blocked aminoacyl tRNA (such as peptidyl tRNA, N-acetylated aminoacyl tRNA, or formyl-methionine tRNA) untouched (Fig. 2A) (23, 28). Thus, in sequencing data, peptidyl tRNA and other N-blocked aminoacyl tRNAs will have intact CCA 3′ ends, whereas aminoacyl tRNAs and deacylated tRNAs will have CC 3′ ends (Fig. 2B).

**FIG 3:**
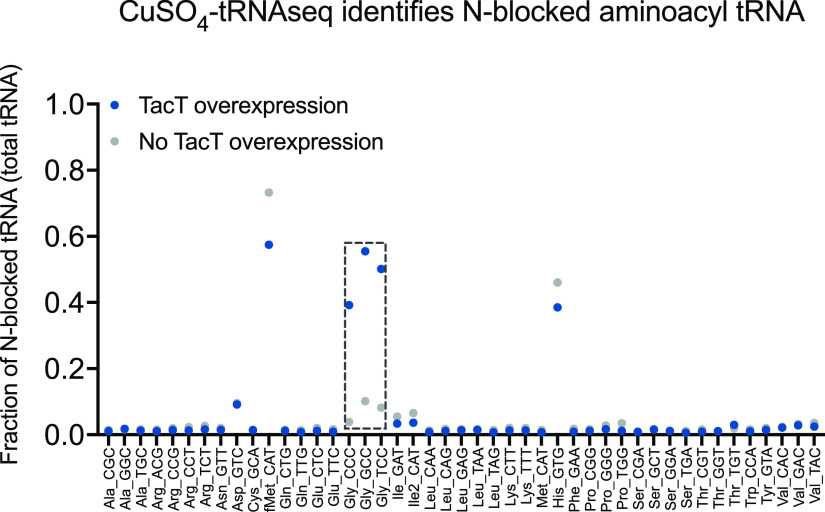
CuSO_4_-tRNA sequencing (Cu-tRNAseq) distinguishes N-acetylated aminoacyl tRNA from aminoacyl or uncharged tRNA. Cu-tRNAseq was carried out on a previously described M. smegmatis (Msmeg) strain with inducible Mtb TacT overexpression (22). The fraction of peptidyl tRNA is plotted as a ratio of the number of 3′ CCA-ending reads to the sum of the 3′ CCA- and 3′ CC-ending reads for each tRNA species (Data Set S1). Cu-tRNAseq detected N-acetylated glycyl-tRNA under induced (TacT overexpression) conditions (dashed box). The results from one biological replicate are shown.

As a proof of principle for the Cu-tRNAseq approach, we used our previously described Mycobacterium smegmatis (Msmeg) strain that overexpresses Mtb TacT, a toxin that adds an acetyl group to the amino acid on charged tRNA (22). Msmeg lacks *tacT*, and since Mtb TacT acetylates glycyl-tRNA, we hypothesized that the protected glycyl-tRNA fraction would increase with TacT overexpression. Indeed, we observed an increased percentage of protected glycyl-tRNA (Fig. 3), suggesting that Cu-tRNAseq can be used to measure N-blocked aminoacyl tRNA. Additional support for this conclusion was the observation that there were relatively high levels of protected initiator tRNA (fMet), which contains a formylated methionine. Histidyl-tRNA was also resistant to CuSO_4_ deacylation, suggesting that histidine’s unique imidazole ring affects the reaction’s efficiency. Together, these observations indicate that Cu-tRNAseq provides a method to distinguish N-blocked aminoacyl tRNAs from aminoacyl tRNAs and therefore should be useful for measuring peptidyl tRNA levels.

### Pth depletion in Mtb reduces pools of usable tRNA.

We applied Cu-tRNAseq to Mtb samples to compare tRNA profiles between strains that were induced for *pth* depletion by CRISPRi and uninduced strains with wild-type Pth levels. The three chemical pretreatments, CuSO_4_, NaIO_4_, and β-elimination, as well as Pth depletion did not appreciably skew tRNA read counts (see Table S1 and Fig. S1 in the supplemental material).

10.1128/mbio.03469-22.1 FIG S1 Chemical treatments do not bias tRNA counts in Cu-tRNAseq. Pairwise linear regression and Pearson’s correlation analysis were performed using the mean tRNA count profiles (see Table S1 in the supplemental material) of different treatment groups. Pearson’s correlation coefficient is depicted on the top left corner of each subpanel. Download FIG S1, EPS file, 2.7 MB. Copyright © 2023 Tomasi et al. 2023 Tomasi et al. https://creativecommons.org/licenses/by/4.0/ This content is distributed under the terms of the Creative Commons Attribution 4.0 International license.

10.1128/mbio.03469-22.5 TABLE S1 MICs of antibiotics in Mtb Pth proteolytic degradation strains. MICs (micrograms per milliliter) are shown for a panel of antibiotics against Pth depletion strains by proteolytic degradation. For the Pth-tagged control, a *pth-flag-DAS* strain was used as a parental backbone for generating knockdown strains (by introducing inducible SspB expression plasmids). This strain serves as a control for the potential effects of the FLAG or DAS tag on Pth activity even in the absence of induced degradation mediated by SspB. Given the leakiness of plasmids and the effects of DAS tagging of Pth even in the absence of SspB, the CRISPRi strain described in the text was used for further phenotypic validation of drug interactions. Download Table S1, EPS file, 2.2 MB. Copyright © 2023 Tomasi et al. 2023 Tomasi et al. https://creativecommons.org/licenses/by/4.0/ This content is distributed under the terms of the Creative Commons Attribution 4.0 International license.

**FIG 4:**
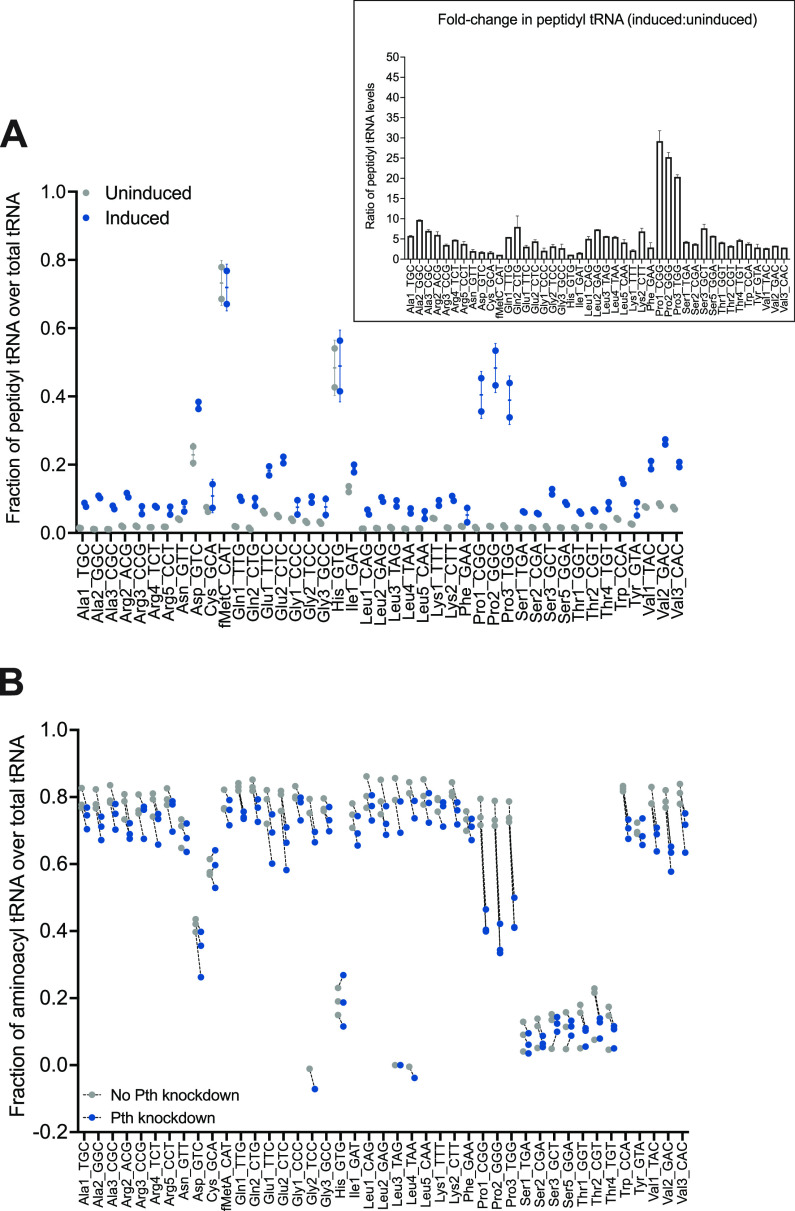
Depletion of Pth leads to the accumulation of peptidyl tRNAs in Mtb and a reduction in usable aminoacyl tRNA pools. Cu-tRNAseq was carried out in an Mtb CRISPRi Pth knockdown construct. (A) Strains were grown to an OD_600_ of 0.3 to 0.4 in the presence or absence of the inducer (50 ng/mL aTC), and total RNA was harvested. The fraction of peptidyl tRNA is calculated as the ratio of the number of 3′ CCA-ending reads to the sum of the 3′ CCA- and 3′ CC-ending reads for each tRNA species. The bar plot at the top right displays the ratios of peptidyl tRNA fractions between induced and uninduced strains. Values from two biological replicates are shown. (B) Mtb RNA samples were treated with periodate and β-elimination only, without CuSO_4_ (see Data Set S2 in the supplemental material), to measure the fraction of the tRNAs protected by either a single amino acid or a peptide. The aminoacyl tRNA fraction for each species is calculated by subtracting the protected fraction with CuSO_4_ treatment from the total protected fraction. Dashed lines connect protected tRNA fractions in uninduced (no Pth knockdown) with those in induced (Pth knockdown) samples to show the overall decrease in usable charged tRNA pools. Values from three biological replicates are shown. Serine and threonine tRNAs are typically acylated at lower levels than other tRNAs in bacteria (30).

Without *pth* depletion, in most tRNA species, a small fraction was protected after copper sulfate treatment, consistent with the idea that, generally, only a tiny fraction of tRNA exists as peptidyl tRNA. However, some tRNA species, such as tRNA-Asp, -fMet, -His, and -Ile, showed a higher proportion of protected species, suggesting that these peptidyl tRNAs accumulated or that these aminoacyl tRNAs are more tolerant to CuSO_4_ treatment (as is the case with fMet). With Pth knockdown, the proportion of tRNAs protected from copper sulfate treatment generally increased (Fig. 4A). The accumulation of protected proline tRNAs was particularly dramatic, suggesting that there is a marked accumulation of peptidyl prolyl-tRNAs in the absence of wild-type Pth levels. Acid-PAGE Northern blotting was used to corroborate that peptidyl prolyl-tRNA accumulates under these conditions (Fig. S2). These results indicate that peptidyl prolyl-tRNAs specifically accumulate when Pth is depleted, suggesting that Pth promotes the robust turnover of peptidyl prolyl-tRNAs in wild-type Mtb cells.

10.1128/mbio.03469-22.2 FIG S2 Northern blotting with arginine and proline tRNA probes. Northern blot assays were performed as described in Materials and Methods. (Top) RNA blotted using a probe for tRNA-Arg_ACG. (Bottom) RNA blotted using a probe for tRNA-Pro_GGG. CuSO_4_ treatment was performed where indicated on total RNA samples as described in Materials and Methods. Alkali treatment, which also deacylates tRNA, was performed on total RNA where indicated (1 h at 37°C in 100 mM Tris [pH 9.0]). Peptidyl, aminoacyl, and deacyl tRNAs are indicated by arrows. Peptidyl prolyl-tRNA is visible under Pth-depleted conditions but not under uninduced conditions. CuSO_4_ treatment deacylates aminoacyl tRNA but not peptidyl tRNA. KD, knockdown. Download FIG S2, EPS file, 1.9 MB. Copyright © 2023 Tomasi et al. 2023 Tomasi et al. https://creativecommons.org/licenses/by/4.0/ This content is distributed under the terms of the Creative Commons Attribution 4.0 International license.

10.1128/mbio.03469-22.7 DATA SET S1 Alignment files for Msmeg Cu-tRNAseq. Msmeg tRNA alignments are provided for all indicated experimental conditions and replicates in each tab. Values represent the frequencies of sequencing read termination at each nucleotide for all tRNA species (read 5′ to 3′), as described in Materials and Methods. Download Data Set S1, XLSX file, 0.1 MB. Copyright © 2023 Tomasi et al. 2023 Tomasi et al. https://creativecommons.org/licenses/by/4.0/ This content is distributed under the terms of the Creative Commons Attribution 4.0 International license.

10.1128/mbio.03469-22.8 DATA SET S2 Alignment files for Mtb Cu-tRNAseq. Mtb tRNA alignments are provided for all indicated experimental conditions and replicates in each tab. Values represent the frequencies of sequencing read termination at each nucleotide for all tRNA species (read 5′ to 3′), as described in Materials and Methods. Download Data Set S2, XLSX file, 0.3 MB. Copyright © 2023 Tomasi et al. 2023 Tomasi et al. https://creativecommons.org/licenses/by/4.0/ This content is distributed under the terms of the Creative Commons Attribution 4.0 International license.

We hypothesized that the accumulation of peptidyl tRNAs would be accompanied by a decrease in aminoacyl tRNA levels. To profile aminoacyl tRNA levels, we conducted tRNAseq with periodate treatment and β-elimination only and measured the ratios of the protected fractions, which together represent the total of the aminoacyl tRNA and peptidyl tRNA fractions. The aminoacyl tRNA proportion was calculated by subtracting the proportion of peptidyl tRNA fractions measured by Cu-tRNAseq from the total charged tRNA fractions, including aminoacylated species (Fig. 4A and B). Prolyl-tRNA availability was drastically decreased under Pth depletion conditions due to the accumulation of peptidyl prolyl-tRNA, indicating that Pth is required for maintaining available prolyl-tRNAs under normal conditions (Fig. 4B).

### Pth depletion increases Mtb sensitivity to candidate aminoacyl tRNA synthetase inhibitors.

**FIG 5:**
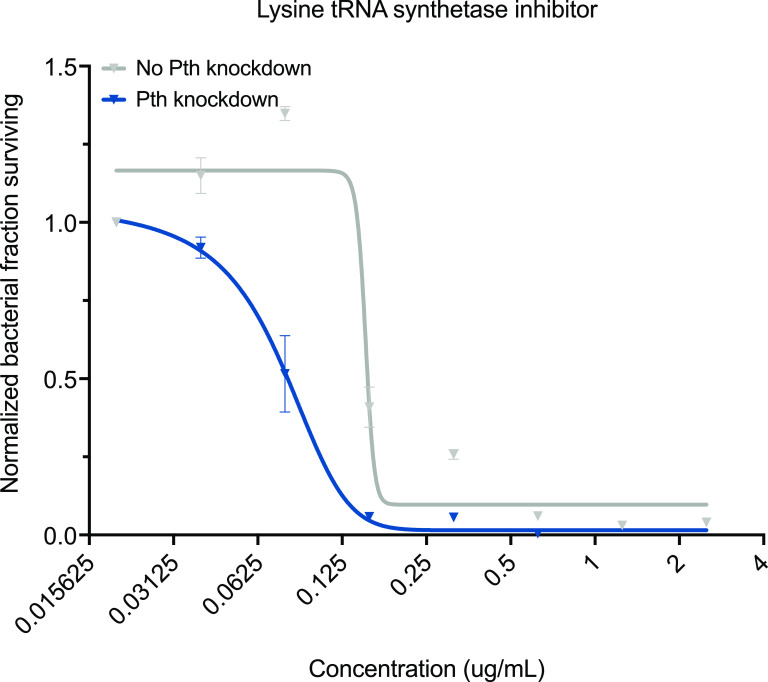
Pth depletion increases Mtb sensitivity to a candidate lysine tRNA synthetase inhibitor. Mtb growth in the presence of a candidate lysine tRNA synthetase inhibitor (compound 7 [31]) is plotted across drug concentrations as determined by fluorescence in an alamarBlue assay. Briefly, antibiotic-containing plates were incubated with Mtb cells for 6 days, at which point resazurin was added, followed by 48 h of additional agitation at 37°C. Fluorescence was normalized to the OD_600_ and to the positive control for each strain (no antibiotic). The fractions of bacteria surviving relative to the no-drug control (“Normalized bacterial fraction surviving”) are plotted against the drug concentrations, along with a least-squares fit of the dose response. Pth CRISPRi strains were grown to mid-log phase, diluted to an OD_600_ of 0.001, and plated with serial dilutions of antibiotics in 96-well plates. The fraction of Mtb cells surviving is plotted by normalizing the fluorescence values to the values for the control wells with no drug, and a least-squares fit of the dose-response data is plotted. Means with standard errors from two biological replicates are shown.

Since the tRNA sequencing findings revealed that Pth promotes tRNA turnover in Mtb, we hypothesized that further targeting a single tRNA species would have a compound effect on Mtb survival in the absence of Pth; i.e., we explored whether limiting tRNA availability by some other mechanism would synergize with Pth depletion. Drugs that inhibit aminoacyl tRNA synthetases have seen clinical success against malaria (halofuginone) and certain skin infections (mupirocin), and efforts are under way to develop similar inhibitors for TB therapeutics. We tested a candidate lysine tRNA synthetase inhibitor (compound 7 [29]) and found a roughly 4-fold decrease in the MIC with Pth knockdowns compared to the uninduced controls (Fig. 5). These observations suggest that orthogonally targeting Mtb’s ability to maintain normal aminoacyl tRNA levels could represent a powerful anti-TB strategy, and we hypothesize that synergy would be particularly potent with a candidate proline tRNA synthetase inhibitor since lysine tRNA levels were less drastically impacted by Pth knockdown than proline tRNA levels.

### Pth depletion sensitizes Mtb to macrolide antibiotics.

**FIG 6:**
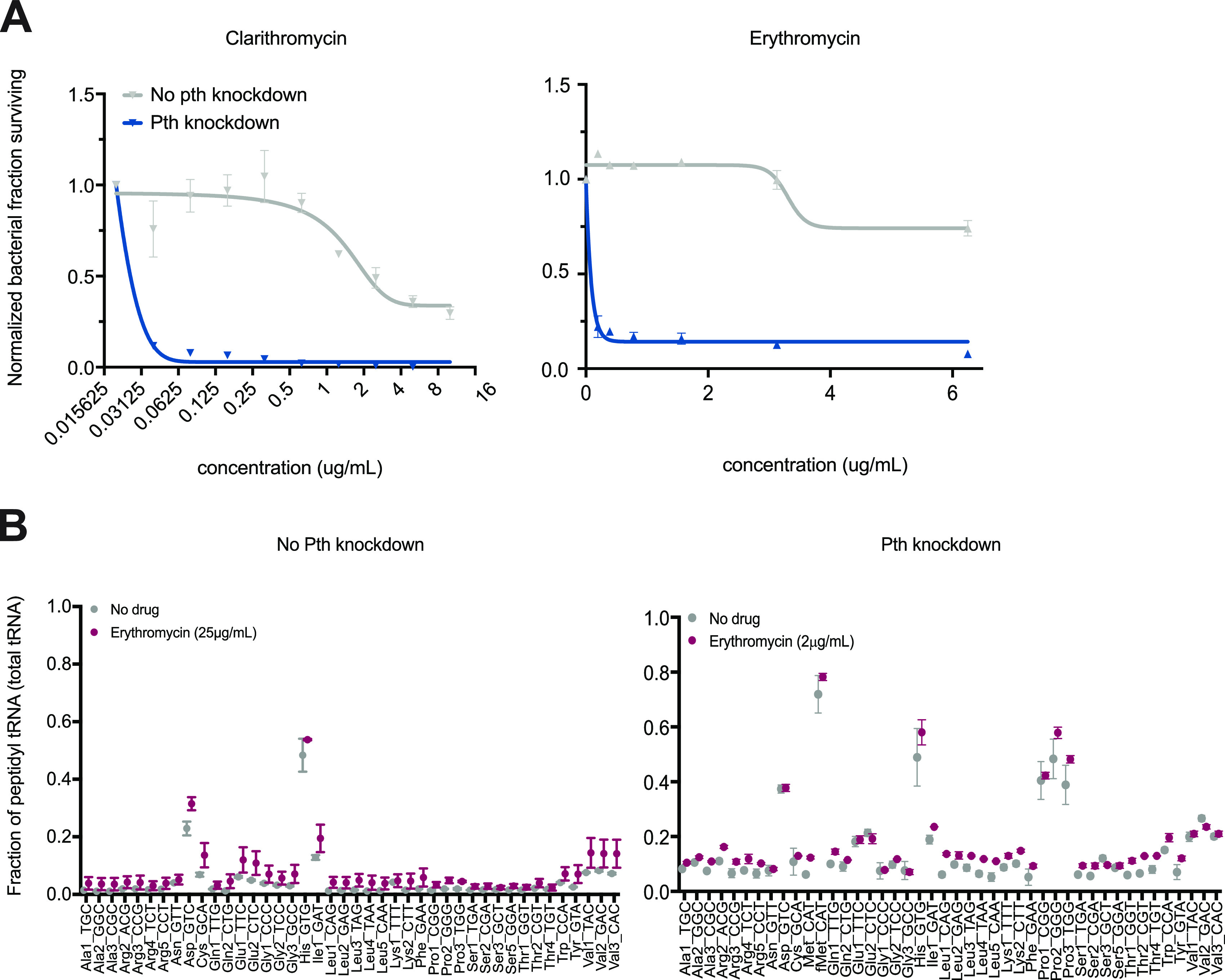
Pth depletion sensitizes Mtb to macrolides. (A) Mtb growth in the presence of two macrolides, erythromycin and clarithromycin, is plotted across drug concentrations as determined by fluorescence in an alamarBlue assay. Briefly, antibiotic-containing plates were incubated with Mtb cells for 6 days, at which point resazurin was added, followed by 48 h of additional agitation at 37°C. Fluorescence was normalized to the OD_600_ and to the positive control for each strain (no antibiotic). The fractions of bacteria surviving relative to the no-drug control (“Normalized fraction surviving”) are plotted against drug concentrations, along with a least-squares fit of the dose response (39). The Pth CRISPRi strain (22) was grown to mid-log phase, diluted to an OD_600_ of 0.001, and plated with serial dilutions of antibiotics in 96-well plates. The fraction of Mtb cells surviving is plotted by normalizing the fluorescence values to the values for the control wells with no drug. Means with standard errors from three biological replicates are shown. (B) Cu-tRNAseq was performed on Pth CRISPRi strains in the presence and absence of erythromycin. Strains were grown in the presence or absence of the inducer as described in the legend of Fig. 3. Uninduced strains were then treated with 25 μg/mL of erythromycin overnight, and Pth knockdown-induced strains were treated with 2 μg/mL for the same duration. Means with standard errors from three biological replicates are shown.

In E. coli, a temperature-sensitive Pth mutant is hypersusceptible to macrolides, a class of antibiotics that target the large 50S ribosomal subunit (30). Macrolides are thought to increase the rates of peptidyl tRNA drop-off by obstructing the nascent peptide exit tunnel, leading to early ribosome dissociation from transcripts (31, 32). Macrolides are not currently used to treat tuberculosis but are generally well tolerated and are used to treat nontuberculous mycobacterial infections (33–35). To explore whether the inhibition of Pth would sensitize Mtb to macrolides, we tested our collection of Pth knockdown strains against a panel of macrolides. Interestingly, all levels of Pth depletion tested rendered Mtb hypersusceptible to macrolides but not to other ribosome-targeting antibiotics that have different mechanisms of action (Fig. 6A; Table S2). Pth hypomorphs were also more susceptible to the lincosamide clindamycin and the aminonucleoside puromycin, both of which also trigger peptidyl tRNA drop-off (Fig. S3) (30, 36). These results suggest that inhibitors that partially block wild-type Pth activity would render Mtb susceptible to treatment with macrolides or other drugs that also increase peptidyl tRNA drop-off.

10.1128/mbio.03469-22.3 FIG S3 Pth depletion increases Mtb susceptibility to clindamycin and puromycin. Mtb growth in the presence of clindamycin (left) or puromycin (right) is plotted across drug concentrations as determined by fluorescence in an alamarBlue assay. Pth CRISPRi strains were grown to mid-log phase, diluted to an OD_600_ of 0.001, and plated with serial dilutions of antibiotics in 96-well plates. The normalized fraction of Mtb cells surviving is plotted by normalizing the fluorescence values to the values for the control wells with no drug (see Materials and Methods). Results are representative of data from two biological replicates. Download FIG S3, EPS file, 1.6 MB. Copyright © 2023 Tomasi et al. 2023 Tomasi et al. https://creativecommons.org/licenses/by/4.0/ This content is distributed under the terms of the Creative Commons Attribution 4.0 International license.

10.1128/mbio.03469-22.6 TABLE S2 Strains, plasmids, and primers used in this work. All strains, plasmids, and primers used for this work are listed. Tab 1 Strains; tab 2 plasmids; tab 3 primers. Download Table S2, XLSX file, 0.01 MB. Copyright © 2023 Tomasi et al. 2023 Tomasi et al. https://creativecommons.org/licenses/by/4.0/ This content is distributed under the terms of the Creative Commons Attribution 4.0 International license.

Indeed, tRNA sequencing shows a slight accumulation in wild-type Mtb cells treated with an inhibitory concentration of erythromycin and a larger increase in peptidyl tRNA pools across tRNAs in a Pth knockdown construct treated with erythromycin (Fig. 6B). The effect on wild-type cells is likely negligible owing to the presence of Pth, but the larger fractions of peptidyl tRNA in the Pth hypomorph suggest that cells are overloaded with Pth substrates upon erythromycin treatment. This effect was not observed for tRNA pools from Mtb cells treated with chloramphenicol, which blocks translation by binding to a different part of the 50S ribosomal subunit and inhibiting peptide bond formation (Fig. S4). These findings confirm that macrolides increase peptidyl tRNA drop-off and that even slightly perturbing the tRNA pools in a Pth knockdown construct leads to significant phenotypic consequences.

10.1128/mbio.03469-22.4 FIG S4 Chloramphenicol does not increase peptidyl tRNA drop-off. Cu-tRNAseq was performed on Mtb strains induced for Pth depletion by CRISPRi in the presence and absence of chloramphenicol (2 μg/mL). After 3 days of incubation with the inducer (50 ng/mL aTC), chloramphenicol was added to the cultures, and the cultures were incubated overnight prior to RNA extraction and sample processing. Chloramphenicol treatment (pink) led to a reduction in peptidyl tRNA pools relative to the drug-free control, suggesting that chloramphenicol-induced protein synthesis arrest affects tRNA pools differently from the other ribosome inhibitors tested in this work. The results are from two biological replicates. Download FIG S4, EPS file, 1.7 MB. Copyright © 2023 Tomasi et al. 2023 Tomasi et al. https://creativecommons.org/licenses/by/4.0/ This content is distributed under the terms of the Creative Commons Attribution 4.0 International license.

## DISCUSSION

In this work, we developed a new application of tRNA sequencing to monitor peptidyl tRNA levels. Cu-tRNAseq builds on existing techniques by incorporating copper sulfate treatment to chemically distinguish peptidyl tRNA from aminoacyl or uncharged tRNA, facilitating the study of the composition of tRNA pools in a quantitative and high-throughput manner. Using Cu-tRNAseq, we found that the depletion of Pth led to reductions in most aminoacyl tRNAs but that proline tRNA pools were the most substantially impacted. Our findings suggest that Pth is particularly important for recycling peptidyl tRNA that has dropped off during the incorporation of proline into a growing peptide in Mtb. Mycobacteria are a highly GC-rich genus, and pathogenic mycobacteria are enriched for proline/glutamate-rich (PE/PPE) proteins, outer membrane proteins characterized by their proline-rich N-terminal sequences that make up nearly 10% of the Mtb genome (37, 38). It is tempting, then, to speculate that Mtb’s proline richness contributes to a critical need for Pth to promote robust proline tRNA turnover and keep up with the cells’ demand for prolyl-tRNA.

Peptidyl tRNA drop-off early in translation is thought to be mediated by the ribosome release and recycling factors RF3 and RRF (6). While the peptidyl tRNA substrates from this mechanism of drop-off have not specifically been characterized prior to this work, recent studies have characterized another phenomenon known as intrinsic ribosome destabilization (IRD), in which a longer nascent chain can also trigger premature translation termination (39). While the mechanism of IRD also requires further study, peptidyl tRNA released into the cytoplasm during abortive IRD can be a substrate for Pth (39). Interestingly, studies of these truncated peptides found a relationship between the presence of alternating proline and acidic amino acids and the likelihood that a ribosome will be destabilized (39). These findings corroborate our findings that proline amino acids predispose ribosomes to abortive translation.

Even though proline tRNA turnover was the most impacted by Pth depletion, we found that Mtb Pth hypomorphs were more susceptible to lysine tRNA synthetase inhibitors than uninduced controls. This suggests that the smaller reductions that we observed in aminoacyl tRNA availability in other tRNAs (where there were lower rates of peptidyl tRNA drop-off) may also be biologically significant. *pth* is essential in many other bacterial species, including E. coli and Bacillus subtilis, neither of which is enriched for polyproline-containing proteins, suggesting that Pth controls tRNA pools more broadly. In fact, studies of an E. coli temperature-sensitive Pth mutant found suppressor mutations that led to the overproduction of lysine tRNA, suggesting that the growth defect in E. coli may be due to lysine tRNA starvation in this AT-rich organism (40). Overexpressing other tRNAs individually, meanwhile, did not rescue the growth defect (40). However, later work found that lysine tRNA overexpression also led to increased protein synthesis, including Pth synthesis, raising questions about the interpretability of the lysine tRNA suppressor and potentially pointing to a more global tRNA effect as in Mtb (41). Future work investigating peptidyl tRNA pools in *pth* mutants in other bacteria would shed light on the dynamics of peptidyl tRNA drop-off and Pth’s role in other organisms. The tRNA sequencing-based methods outlined in this paper provide a scalable and efficient platform for such efforts.

Tuberculosis (TB) remains a leading infectious disease threat to global health. Mtb is intrinsically resistant to most existing antibiotics, and current treatment requires a minimum of 4 months of combination therapy using potent drug regimens, which have remained largely unchanged since their discovery in the mid-20th century. Protein synthesis is indispensable to any living cell, and in fact, many of the antibiotics used to treat other bacterial infectious diseases target some aspect of protein synthesis. Nonetheless, most of these drugs are ineffective against TB, and our current anti-TB arsenal is lacking in protein synthesis inhibitors. Here, we have identified several orthogonal approaches to inhibit protein synthesis in Mtb by impeding an essential mediator of tRNA availability. Our work underscores Pth as a promising candidate for target-based development given its stand-alone requirement for Mtb survival as well as its ability to potentiate the activities of two classes of translation inhibitors.

Macrolides are used to treat a variety of pathogens, including some nontuberculous mycobacteria, but are currently ineffective against TB. Sensitizing Mtb to macrolides by simultaneously targeting Pth offers a new strategy to lend efficacy to an entire class of antibiotics against this devastating disease. Many mycobacteria encode an inducible macrolide resistance mechanism (42, 43). Exposure to macrolides triggers the expression of the so-called *erm* family of genes, whose products methylate ribosomes at the site of macrolide binding. Despite possessing this innate resistance mechanism, macrolides like azithromycin are still used to treat Mycobacterium abscessus infections, suggesting that there could still be success in including macrolides in an anti-TB regimen (33). Since Pth depletion causes growth arrest, we hypothesize that treating Pth-depleted cells with macrolides will make it difficult, if not impossible, for cells to actively synthesize Erm methyltransferases, whose synthesis is induced by the WhiB7 intrinsic-resistance transcription factor, further contributing to their sensitivity to macrolide treatment (44, 45). Furthermore, Mtb clinical isolates lacking *erm* genes have been discovered in an Mtb sublineage that is endemic to Southeast Asia, and multiple other macrolide-sensitizing genes have been identified in Mtb and are worth exploring as additional adjuvants in a macrolide-based anti-TB strategy (44).

A recent survey of gene vulnerability to various levels of depletion using CRISPR interference (CRISPRi) found that even slightly reducing the levels of tRNA synthetase expression causes major growth attenuation in Mtb (46). Inhibitors of these enzymes are currently being developed, with a focus on lysine tRNA synthetases. Our work suggests that there is an opportunity to strengthen these already highly potent compounds even further by coupling them with an orthogonal regulator of tRNA availability. While existing proline tRNA synthetase inhibitors like halofuginone unfortunately do not exhibit activity against Mtb *in vitro*, our findings raise the question of whether a Pth inhibitor would synergize more strongly with proline tRNA synthetase inhibitors given the disproportionate decrease in proline tRNA availability in a Pth knockdown construct.

Altogether, our work underscores the importance of Pth in tRNA turnover and provides new biological insights into anti-TB strategies that could exploit the synergy between translation errors and tRNA turnover. We also demonstrate a new application of tRNA sequencing that can be used to study peptidyl tRNAs and N-acetylated tRNAs in other organisms, providing a high-throughput way to shed light on the dynamics of tRNA pools across all kingdoms of life.

## MATERIALS AND METHODS

### Bacterial strains and growth conditions.

Mtb and Msmeg strains were grown from frozen stocks in Middlebrook 7H9 medium supplemented with 0.2% glycerol, 0.05% Tween 80, and albumin-dextrose-catalase (ADC) (5 g/L bovine serum albumin, 2 g/L dextrose, 3 μg/mL catalase) with and without oleic acid, respectively. Cultures were incubated at 37°C. The following antibiotics or inducing agents were used for both Mtb and Msmeg when needed: kanamycin (25 μg/mL), anhydrous tetracycline (aTC) (100 ng/mL), hygromycin (50 μg/mL), and nourseothricin (20 μg/mL). Transformed Mtb and Msmeg strains were plated onto 7H10 agar plates with the appropriate antibiotic(s). Strains were grown to mid-log phase for all experiments unless otherwise specified (optical density at 600 nm [OD_600_] of 0.4 to 0.6). E. coli strains for cloning or protein purification were grown in LB broth or on LB agar with the following appropriate antibiotics: kanamycin (50 μg/mL), zeocin (50 μg/mL), and nourseothricin (40 μg/mL). The induction time for *pth* depletion in Mtb was 3 days.

### Bacterial strain construction.

Table S2 in the supplemental material depicts the strains, plasmids, primers, and recombinant DNA used for this study. Plasmids were built by restriction digestion of a parental vector, and inserts were prepared by either restriction enzyme cloning or Gibson assembly using 40-bp overhangs, as specified in Table S2. Plasmids were isolated from E. coli and confirmed via Sanger sequencing carried out by Genewiz, LLC (MA, USA).

### Pth knockdown constructs.

Transcriptional knockdown of *pth* was accomplished using mycobacterial CRISPR interference (CRISPRi) as described previously (22). Proteolytic degradation strains for degradation using a titratable system (20) were built using the plasmids and primers listed in Table S2.

### Northern blotting.

Acid-PAGE Northern blotting of tRNA was performed as previously described (47). Briefly, 0.5 μg total RNA was electrophoresed on a 6.5% urea gel (7 M urea, 100 mM sodium acetate [NaOAc] [pH 5.0]), transferred to a nitrocellulose membrane by semidry blotting, and UV cross-linked twice (1,200 μJ). Membranes were incubated with ULTRAhyb-oligo (Thermo Fisher Scientific) at 42°C for 30 min, followed by hybridization overnight at 42°C with 4 pmol probes (Table S2) that were radiolabeled using [γ-^32^P]ATP (PerkinElmer) and T4 polynucleotide kinase (New England BioLabs). Membranes were washed twice with 2× SSC (1× SSC is 0.15 M NaCl plus 0.015 M sodium citrate) plus 0.1% SDS, and the bound probe was detected using an FLA-5000 PhosphorImager (Fuji).

### tRNA sequencing.

**(i) Extraction of total RNA.** Strains were grown to mid-log phase with the appropriate antibiotics and inducing agents described above. RNA was collected at the same OD_600_ for each strain (between 0.4 and 0.6). Cells were left on ice for 20 min and then pelleted by centrifugation at 4,000 rpm for 10 min at 4°C. Pellets were resuspended in 0.5 to 1 mL of TRIzol (Life Technologies) and lysed using a BeadBug microtube homogenizer (MilliporeSigma). Two hundred microliters of chloroform was added to each tube, after which samples obtained from Mtb strains were removed from biosafety level 3 precautions. Samples were centrifuged at 15,000 rpm for 15 min at 4°C, and the aqueous layer was collected into a fresh tube. Two hundred fifty microliters of sodium acetate buffer (300 mM sodium acetate [pH 5.2] and 10 mM EDTA [pH 8.0]) was added to the original tube, and samples were vortexed at 4°C for 5 min and then centrifuged at 15,000 × *g* for 15 min at 4°C. The aqueous layer was added to the fresh sample-containing tubes. Four hundred microliters of chloroform was added, and the tubes were briefly vortexed and then centrifuged at 15,000 rpm for 1 min at 4°C. The aqueous phase was collected into a fresh tube, and RNA was recovered by ethanol precipitation. RNA pellets were resuspended in 10 mM sodium acetate (pH 5.2) and stored at −80°C until they were processed for sequencing.

**(ii) Copper sulfate treatment.** Copper sulfate (CuSO_4_) treatment of total RNA samples was performed as described previously (23). Briefly, 20 to 30 μg of total RNA was added to fresh tubes to a final volume of 54 μL in storage buffer (8 M urea, 10 mM sodium acetate [pH 5.2], 1 mM EDTA). Six microliters of 100 mM CuSO_4_ was added to a final concentration of 10 mM CuSO_4_, and reaction mixtures were incubated at 37°C for 1 h. A total of 1 mM EDTA was then added to each tube, along with 3 μL of glycogen (2 mg/mL). Samples were recovered by ethanol precipitation and stored at −80°C between treatments.

**(iii) Sodium periodate treatment.** Sodium periodate (NaIO_4_) treatment of total RNA samples was performed as described previously (26). Briefly, 1 μg of total RNA was combined with 100 mM sodium acetate (pH 5.2) and 50 mM NaIO_4_ in a final volume of 100 μL. Reaction mixtures were incubated at room temperature in the dark for 30 min and subsequently quenched with 100 mM glucose for 5 min. Unquenched periodate was removed using Micro Bio-Spin P-6 columns (Bio-Rad Laboratories) according to the manufacturer’s instructions. RNA was recovered by ethanol precipitation.

**(iv) β-Elimination treatment.** β-Elimination of periodate-oxidized RNA samples was performed as described previously (26). Briefly, total RNA collected after periodate oxidation was combined with 60 mM sodium borate (pH 9.5) (Boston BioProducts) in a final volume of 100 μL and incubated for 90 min at 45°C. Samples were purified with Micro Bio-Spin P-6 columns (Bio-Rad Laboratories) according to the manufacturer’s instructions. RNA was recovered by ethanol precipitation.

### Library preparation.

**(i) Isolation of tRNA fractions.** A total of 1 to 2 μg of total RNA was run on a 10% Tris-borate-EDTA (TBE)–urea gel (Thermo Fisher Scientific) at 250 V for 1 h. Gels were stained with SYBR gold (Thermo Fisher Scientific), and tRNA was excised. Excised gels containing tRNA fractions were mashed in RNase-free tubes, and 300 μL elution buffer (300 mM NaOAc [pH 5.5], 1 mM EDTA [pH 8.0], 0.10% SDS) was added to each tube. Samples were shaken on a thermoshaker (VWR) for 1 to 4 h at 37°C, and the supernatant was collected using an Ultrafree filter column (MilliporeSigma). tRNA was recovered by isopropanol precipitation.

**(ii) tRNA dephosphorylation.** tRNA was dephosphorylated using QuickCIP (New England BioLabs) according to the manufacturer’s instructions, and tRNA was collected by phenol-chloroform extraction followed by isopropanol precipitation.

**(iii) Adapter ligation.** A total of 0.5 μL RNase inhibitor was added to 3.5 μL dephosphorylated tRNA (200 to 250 ng tRNA), and samples were boiled at 80°C for 2 min. Boiled tRNA was mixed with 12 μL polyethylene glycol (PEG) buffer mix (10 μL 50% PEG 8000, 2 μL 10× buffer B0216S [New England BioLabs]). Three microliters of 5′-adenylated linkers (Table S2) was added (33 pmol/μL) along with 1 μL truncated T4 RNA ligase 2 (New England BioLabs), and the mixture was incubated at 25°C for 2.5 h. Samples were recovered by isopropanol precipitation and run on a 10% TBE-urea-PAGE gel for 40 min at 250 V. Ligated products were recovered by gel excision as described above.

**(iv) Reverse transcription.** Identical quantities of samples with different adapter sequences were pooled for reverse transcription for a total of 200 to 250 ng tRNA. Reverse transcription was performed by combining 2.1 μL dephosphorylated tRNA with a solution containing 100 mM Tris-HCl (pH 7.5), 0.5 mM EDTA, 1.25 μM reverse transcriptase (RT) primer (Table S2), 450 mM NaCl, 5 mM MgCl_2_, 5 mM dithiothreitol (DTT), 500 nM thermostable group II intron RT (TGIRT) (InGex), and 15% PEG 8000 in a final volume of 9 μL. Samples were incubated at 25°C for 30 min, after which 1 μL 10 mM deoxynucleoside triphosphates (dNTPs) (New England BioLabs) was added, and the reaction mixtures were incubated at 60°C for 1 h. A total of 1.15 μL NaOH was added, and samples were boiled for 15 min and run on a 10% TBE-urea-PAGE gel at 250 V for 1 h. Reverse transcription products were excised, and cDNA was recovered by isopropanol precipitation. Linear single-stranded cDNA was circularized using CircLigase II (Lucigen) according to the manufacturer’s instructions.

**(v) PCR of tRNA libraries.** PCR mixtures were set up using HF Phusion according to the manufacturer’s instructions using a universal reverse primer (Table S2) and a different index primer for each pool of samples. PCR mixtures were aliquoted into 4 tubes and collected after 6, 8, 10, and 12 cycles. Samples were run on a native TBE-PAGE gel (Thermo Fisher Scientific) at 180 V for 50 min, and amplified products were cut from the same cycle for each sequencing run. Samples were recovered by gel excision and isopropanol precipitation.

**(vi) Sequencing.** Sequencing was performed on a MiSeq instrument (Illumina) using 150-bp single-end reads with a version 3 150-cycle kit.

**(vii) Analysis.** The 3′ linker sequences and 2 nucleotides at the 5′ end were trimmed using cutadapt and fastx-trimmer. Bowtie v1.2.2 was used with default settings to map reads to reference Mtb or Msmeg tRNA sequences (Data Sets S1 and S2) retrieved from Mycobrowser (48). mpileup files were generated using SAMtools (SAMtools mpileup -I -A –ff 4 -x -B -q 0 -d 10000000). The 3′ ends of mapped reads were piled up using the bedtools genomecov command (-d -3 -ibam). To analyze 3′ ends, a cutoff of 500 read counts for each tRNA species was set. The number of 3′ termini at any position was divided by the total number of mapped termini to compute the 3′ termination frequency. The ratio of peptidyl tRNA for each tRNA species is the ratio of the 3′ termination frequency at the terminal A residue of the 3′ CCA tail of each tRNA sequence to the sum of the frequencies at the terminal A and the previous C nucleotide.

### Drug susceptibility assays.

Drug susceptibility was measured using an MIC assay as described previously (49). Briefly, strains were diluted to an OD_600_ of 0.001 and tested in technical duplicate using serial dilutions of the following antibiotics: erythromycin (GoldBio), clarithromycin (GoldBio), puromycin (GoldBio), clindamycin (GoldBio), chloramphenicol (Sigma-Aldrich), isoniazid (Sigma-Aldrich), ethambutol (Sigma-Aldrich), kanamycin (IBI Scientific), fusidic acid (Sigma-Aldrich), linezolid (Sigma-Aldrich), and capreomycin (Sigma-Aldrich). The lysine tRNA inhibitor (compound 7 [31]) was synthesized at the University of Dundee and dissolved in dimethyl sulfoxide (DMSO). When needed (for example, for *pth* knockdown and empty guide RNA controls), strains were preinduced with aTC as described above. Each MIC assay was conducted in biological triplicates, with each biological replicate conducted in technical duplicate. In plates containing CRISPRi-induced cells, aTC was added to each well at a final concentration of 100 ng/mL. Here, a technical replicate is considered a single row of drug and bacterial incubation, using bacteria from the same culture. Biological replicates are considered plates set up in the same way using bacteria from a different culture. The 96-well plates were agitated at 37°C for 6 days, at which point 0.0002% resazurin was added, and the plates were agitated at 37°C for an additional 48 h. The plates were read on a BioTek plate reader at both 24 h and 48 h. Resazurin was measured by the OD_570_, and the OD_600_ was also measured for each well. Fluorescence was normalized to the OD_600_ and to the positive control for each strain (no drug; in CRISPRi-induced wells, the positive control still contained aTC), and the fraction of bacteria surviving relative to the no-drug control well was plotted against the drug concentration for each well.
